# Total Body Irradiation with Step Translation and Dynamic Field Matching

**DOI:** 10.1155/2013/216034

**Published:** 2013-07-01

**Authors:** Ho-Hsing Chen, Jay Wu, Keh-Shih Chuang, Jia-Fu Lin, Jia-Cheng Lee, Jin-Ching Lin

**Affiliations:** ^1^Department of Radiation Oncology, Taichung Veterans General Hospital, 1650 Taiwan Boulevard Sect. 4, Taichung 40705, Taiwan; ^2^Department of Biomedical Engineering and Environmental Sciences, National Tsing Hua University, Taiwan; ^3^Department of Biomedical Imaging and Radiological Science, China Medical University, Taichung 40402, Taiwan

## Abstract

The purpose of this study is to develop a total body irradiation technique that does not require additional devices or sophisticated processes to overcome the space limitation of a small treatment room. The technique aims to deliver a uniform dose to the entire body while keeping the lung dose within the tolerance level. The technique treats the patient lying on the floor anteriorly and posteriorly. For each AP/PA treatment, two complementary fields with dynamic field edges are matched over an overlapped region defined by the marks on the body surface. A compensator, a spoiler, and lung shielding blocks were used during the treatment. Moreover, electron beams were used to further boost the chest wall around the lungs. The technique was validated in a RANDO phantom using GAFCHROMIC films. Dose ratios at different body sites along the midline ranged from 0.945 to 1.076. The dose variation in the AP direction ranged from 96.0% to 104.6%. The dose distribution in the overlapped region ranged from 98.5% to 102.8%. Lateral dose profiles at abdomen and head revealed 109.8% and 111.7% high doses, respectively, at the body edges. The results confirmed that the technique is capable of delivering a uniform dose distribution to the midline of the body in a small treatment room while keeping the lung dose within the tolerance level.

## 1. Introduction

Total body irradiation (TBI) is a type of external beam radiotherapy. It has been used in conjunction with chemotherapy to prepare patients for bone marrow transplantation (BMT) [1, 2]. A uniform dose distribution throughout the entire body during TBI is necessary to suppress immunological rejection in the recipient and to eliminate residual malignant cells. Therefore, dose impact from irregular body contour and internal tissue heterogeneity must be considered to minimize dose variation within the body [1, 3]. 

Parallel-opposed anterior/posterior (AP/PA) and bilateral (LAT) fields are commonly used in the conventional TBI treatment [4, 5]. For the AP/PA treatment, the patient stands in front of the wall opposite to the treatment head and is irradiated with a large treatment field. Major advantages of the AP/PA treatment are less thickness variation of the body in the superior-inferior direction and reduced radiation dose to the lungs. However, patients might find standing during treatment uncomfortable. Beams delivered bilaterally with patient sitting on the bed are more comfortable, but large thickness variation of the body requires custom designed compensators for individual patient. Shielding the lungs in the LAT position is also a challenge. It is not sufficient to reduce the lung dose by arms only [6]. Additional shieldings are required. However, boosting dose to the tissues surrounding the lungs is technically difficult. From dosimetric perspective, a treatment planning system commissioned under standard treatment condition cannot be used directly for irradiation at an extended treatment distance. Extra scatter from the floor and the wall should be considered [7–9].

Various techniques have been developed for performing TBI that deliver a uniform dose distribution. Chui et al. proposed an arc treatment with a gravity-oriented compensator to deliver a uniform dose to a patient lying on the floor [10]. This method can be implemented in a small treatment room, but it also results in a large penumbra around the lung shielding area. The translating couch technique moves a patient horizontally beneath a vertical beam to achieve a uniform dose distribution. However, a moving couch with adjustable speed is required [11–16]. Recently, sequential beam delivery techniques have been used in TBI. Helical TomoTherapy can continually deliver a uniform dose to a patient on the treatment couch with 360° spiral gantry rotation. This can be achieved using the standard planning beam model without extra management [17–20]. In addition, linear accelerator-based intensity modulated techniques have been used to treat a large target volume with multiple isocenters under the standard treatment condition [21, 22]. The advanced field-in-field (FIF) technique uses a simple method to compensate for body contour variation with lateral beam delivery [23]. The modulated-arc total body irradiation (MATBI) technique delivers a uniform dose to the entire body by rotating gantry fields planned inversely by a new beam model commissioned at an extended source-to-surface distance [24, 25]. The aperture-modulated translating bed TBI (AMTBI) technique synchronizes the aperture with bed motion to improve dose uniformity and reduce dose to the lungs [13, 14]. 

The choice of TBI techniques depends on the clinical requirements, equipments availability, and practicality. In a limited treatment space, it is important to deliver a uniform dose to the entire body without extra equipment or complicated techniques while keeping the lung dose within the tolerance level. 

This study presents a novel step translation dynamic field-matching (STDFM) technique to implement the TBI treatment in a small treatment room with AP/PA beams. Patients can be treated in a comfortable position without complicated bed translation, gantry rotation, and beam modulation. In addition, a uniform dose is delivered to the body, while sparing the lungs. The proposed method was verified by phantom measurement using GAFCHROMIC films. 

## 2. Materials and Methods

 A RANDO phantom was set up on the floor in supine and prone positions for the AP and PA treatments, respectively. For each AP and PA treatment, two oblique fields irradiated the superior and the inferior parts of the body. These two fields were angled obliquely so that the inferior edge of the superior field matched the superior edge of the inferior field. Moreover, through dynamic leaf motion, the matching edges were feathered over an overlapped region marked on the phantom surface. In between the superior and inferior irradiations, the phantom was translated according to these marks. The optimal overlap widths of different leaf motion lengths were investigated and used for the treatment setup. A compensator was used to modulate the slanted beam intensity due to oblique incidence of the fields. A beam spoiler was used to increase the dose in the buildup region. It also served as a platform on which the lung shielding blocks were placed. The lung shielding blocks reduced the lung dose. An additional electron beam was used to boost the dose to the chest wall. 

### 2.1. Dynamic Field Matching

**Figure 1:**
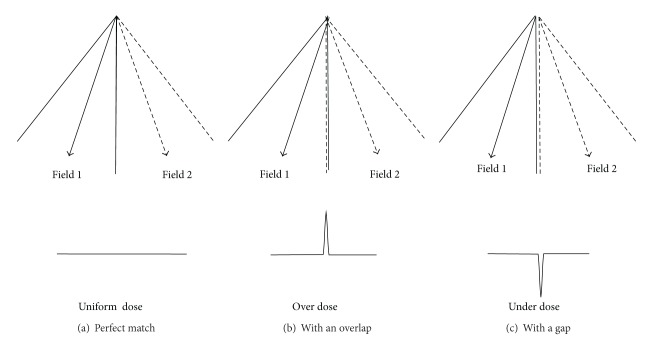
Two adjacent abutting fields. The setup and machine errors cause dose variation where the fields meet. (a) If the two fields are matched perfectly, they produce a uniform dose distribution at the junction. (b) When the fields overlap, an overdose is seen in the dose profile. (c) When there is a gap, an underdose is produced.

 For TBI involving multiple matching fields, dose heterogeneity in the junction region [3] is a major concern. Ideally, fields abutting perfectly at the match line can provide uniform dose across the junction region. In practice, dose variation is usually observed due to setup and machine errors.  Figure 1(a) illustrates an ideal case of perfect field matching which results in a uniform dose across the junction region.  Figure 1(b) shows a slight overlap of the matching fields which produces a significant dose peak in the profile. Similarly, Figure 1(c) shows a gap between the matching fields which results in a cold spot. Magnitude of the dose variation depends on the magnitude of the matching error. 

**Figure 2:**
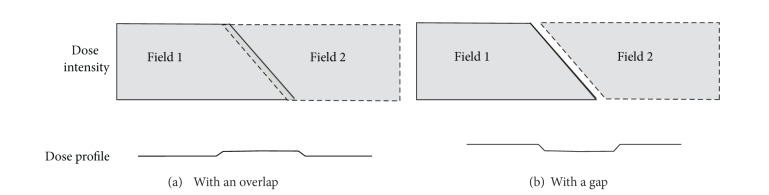
The dynamic field-edge matching technique generates a match zone by overlapping two fields along their border. Two complementary inclined fields smear the dose variations over a wide region. The field intensity profiles are shown with the fields (a) overlapping or (b) separated by a gap.

To deal with dose heterogeneity, a dynamic field-edge matching technique [26–28] that smears dose inhomogeneity over the field matching zone by two complementary inclined fields was used (Figure 2). In order to keep homogeneous junction dose at all depths, the matching field edges must be parallel to each other. Hence, the gantry was rotated according to the beam divergent angle to make the matching field edges aligned. 

 Based on the dynamic field-edge matching technique, a patient was set up on the floor in supine and prone positions. Two oblique fields with an overlapped region on the body surface were delivered by translating the patient to align the matching line with the respective dynamic field edges. As a result, a large volume can be irradiated. 

### 2.2. Dynamic MLC Field Editing

**Figure 3:**
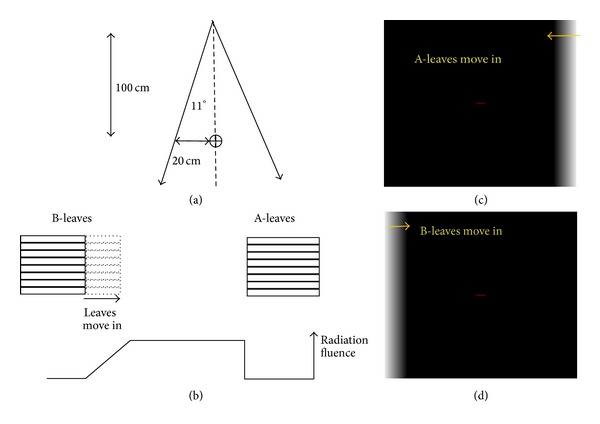
Dynamic MLC fields consist of inclined radiation field. (a) The field geometry is shown. (b) The radiation fluence delivered by a dynamic field. When the beam is on, the leaves move continuously from the 20 cm position to the 17 cm position. (c) The fluence map of the inferior field when the A-leaves move in. (d) The fluence map of the superior field when the B-leaves move in.

 The method of editing dynamic MLC leaf sequence files has been published previously [29]. For this study, the dynamic MLC fields were edited using the Shape Editor (Version 6.1, Varian Medical Systems, Palo Alto, CA, USA) to form a tapered field edge with the fluence decreasing gradually from the value in field to zero at the field edge. The superior dynamic field irradiated the upper part of the body with the B-leaves in motion. It consisted of two segments. The leaves of the first segment were set at the start position with dose fraction 0, and the leaves of the second segment were set at the stop position with dose fraction 1. The A-leaves were fixed at 20 cm. Similarly, the inferior dynamic field irradiating the lower part of the body was created with the A-leaves in motion. Crucially, these two dynamic fields must have the same leaf motion length. With this condition, the two adjacent inclined fields were matched complementarily to produce a uniform dose distribution in the overlapped region.  Figure 3 shows the fluence distribution of the dynamic fields with the leaves moving from location 20 cm to location 17 cm.

### 2.3. Treatment Setup

**Figure 4:**
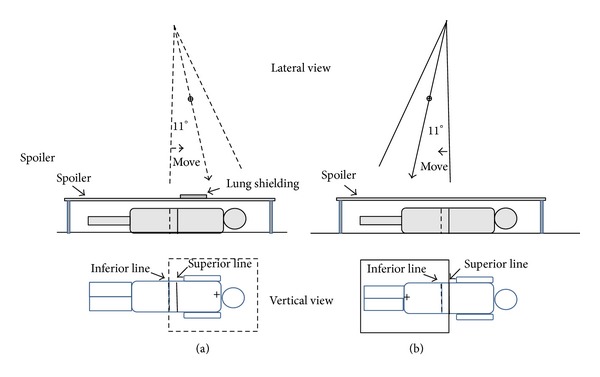
The setup for two-step translation dynamic field-edge matching TBI. (a) The superior field irradiates the upper body with the B-leaves moving in. The dynamic field edge is aligned with the inferior line at a gantry angle of 349° ( = 360° − 11°). (b) After shifting the patient, the inferior field irradiates the lower body with the field edge of the A-leaves aligned with the superior line at a gantry angle of 11°. *⊕* indicates the isocenter of the linear accelerator. + indicates the central axis of the dynamic field.

Figure 4 shows the STDFM TBI treatment setup. A RANDO phantom was set up on the floor in the supine and prone positions beneath the gantry. A beam spoiler was placed 30 cm above the floor. The lung blocks were placed on the spoiler to shield the lung during the superior field irradiation. The distance from the source to the floor was 230 cm.

TBI was performed using two dynamic edge matching fields for each AP/PA treatment. The superior field was used to treat the upper part of the body and the inferior field for the lower part. Between delivery of these two fields, the phantom was translated so that these two fields covered the whole body with an overlap. In order to keep the abutting field edges parallel to each other at all depths, the gantry angle was rotated 11° clockwise for the inferior field and counterclockwise for the superior field. The 11° angle was calculated as tan^−1^(20/100). The leaves were set at the start position of 20 cm measured at a source-to-isocenter distance of 100 cm. All treatments were delivered on a Varian 21EX linear accelerator with a 6 MV photon beam at 40 × 40 cm^2^ field size and 0° collimator angle.

The procedure of the two-step translation is illustrated in Figure 4. The width between the two match lines depends on the leaf motion length of the dynamic fields and is described below. The location of the overlapped region was determined by simulating the treatment conditions of the superior and inferior fields, such that the combined dynamic fields covered the entire body.

In the AP treatment, the phantom was set in the supine position. First, for the superior field, the gantry was set to 349°. The phantom was then translated to align the inferior match line with the inferior edge of the field. The beam was then turned on with the B-leaves set in motion during beam on. After completing delivery of the superior field, the gantry was rotated clockwise to 11° for delivery of the inferior field. The phantom was translated to align the superior matching line with the superior edge of the field. The beam was then turned on with the A-leaves set in motion during beam on. Using this two-step translation, the AP treatment can be delivered via the two dynamic fields. Similarly, for the PA treatment, the phantom was set in the prone position, and the same procedure was repeated as in the AP treatment.

### 2.4. Beam Intensity Compensator

 As described above, in order to match the abutting field edges at all depths, the center lines of the two dynamic fields were incident obliquely at the body surface so that the matching edges were parallel. An oblique incident beam, however, produced a slanted dose profile. An NE-2581 farmer type ionization chamber inserted inside a 20 cm thick solid water phantom (Gammex RMI 457) at a depth of 10 cm was used to measure the dose profile at the treatment distance. Setting the gantry angle at 11° with a 40 × 40 cm^2^ static field size, the transverse dose profiles were measured with the phantom moving along the transverse direction. To modify the oblique beam fluence distribution, a beam compensator composed of a lead sheet of 1 mm in thickness was employed according to the shape of the measured dose profile. The beam compensator was placed on the blocking tray 65 cm from the source. It comprised of two parts: 12 cm width with the lead sheet and 14 cm width without the lead sheet. The transmission factor of the lead sheet was measured under the treatment conditions. Similarly, the dose profile at 10 cm depth was measured with the beam compensator mounted on the accessory mount in the same way.

### 2.5. Optimal Overlapping Widths in the Phantom

 Dose uniformity within the field-matching zone depends on the length of leaf motion and the width of field overlap. Basically, longer leaf motion produces a slower dose gradient and a wider inclined region at the field border. Hence, a wider overlapped region is preferred for better dose uniformity. To determine the optimal overlap widths for various dynamic fields, we created leaf motion lengths of 1-, 2-, 3-, 4-, and 5-cm by the Shaper Editor. The dynamic beam profiles at field borders were measured using the Profiler (Sun Nuclear 1170). 

The dose profiles were measured using the Profiler placed inside a solid water phantom on the floor at 10 cm depth with the gantry angle at 349° for a 40 × 40 cm^2^ and 6MV photon beams. Before the measurements, the profiler was calibrated under the treatment condition. To avoid electronic circuit damage, the dynamic field edge was aligned to detector No. 9 to keep the irradiated area away from the electronic circuit.

The measured dose profiles were exported as text files and normalized to the detector No. 40 located away from the region of leaf motion. To obtain total dose distribution in the junction area of the two dynamic fields, complementary dose profiles were created by reversing the measured dose profiles in position. Summing the measured and created dose profiles assuming different overlap widths, dose distributions in the junction region were obtained. ±10% dose variation criteria were used to screen for the optimal overlap width.

### 2.6. Dose Profiles in the Overlapped Region at Different Depths

 Static parallel matching field edges produce a constant overlap width at all depths. However, dynamic field matching with a changing field edge during beam delivery results in variable overlap widths at different depths. Hence, dose uniformity will vary with depth. To ensure uniform dose distributions in the junction area at all depths, 1.5 cm EBT3 GAFCHROMIC film strips (ISP Corp., Wayne, NJ) was sandwiched between the solid water phantom slabs on the floor, at depths of 0, 5, 10, and 15 cm to measure the total dose profiles in the overlapped region. 

The irradiated films were stored in light-tight bags and scanned 24 h later using an Epson 1680 flatbed scanner with the 48 bit RGB color transmission mode, 72 dpi resolution, and no color correction. The images were saved in the tagged image file format (TIFF), and only the red channel signals were used in subsequent readout procedures. Calibration curve fitting and signal-to-dose conversion were performed using the FilmQA software.

### 2.7. Total Dose Profile of the Dynamic Matching Fields

 The midline dose from the two overlapping dynamic fields should be verified in the phantom with a beam compensator in place to ensure a uniform dose delivery. An ionization chamber (NE 2581) setup under the treatment condition in a 20 cm thick solid water phantom was used to measure the dose profiles at depths of 5, 10, and 15 cm. Two oblique fields, a superior field with the gantry angle set at 349° and an inferior field with the gantry angle set at 11°, delivered the dose to the phantom. Each field has a dynamic edge formed by 3 cm leaf motion, the superior field with the B-leaves moving in while the inferior field with the A-leaves moving in, which produced a 5.5 cm overlapped region on the phantom surface based on the optimal overlap width. To measure the midline dose, the superior and inferior matching lines were drawn on the phantom surface 5.5 cm apart in parallel for measurement setup. The center of the overlapped region was the center of the entire radiation field. During the measurement, the phantom was moved along the transverse axis of the beam, and the ionization chamber accumulated the doses delivered by the two matching fields. The chamber readings were normalized to the reading at the 40 cm position away from the center of the overlapped region. 

### 2.8. Percentage Depth Dose in the Buildup Region

A 1 cm thick acrylic beam spoiler was placed 30 cm above the floor over the phantom. The spoiler-to-phantom distance depends on the thickness of the phantom, for example, 10 cm for a 20 cm thick phantom. Without the spoiler, dose deficiency in the buildup region for megavoltage photon beams results in dose inhomogeneity in the TBI treatment. With the spoiler, electrons scattered out from the spoiler increase the surface dose to near the maximum dose. A Markus parallel plate ionization chamber inserted inside a 20 cm thick solid water phantom was used to measure percentage depth doses in the buildup region for a vertical beam with and without the beam spoiler in place and for an oblique beam with the beam spoiler at the treatment distance.

### 2.9. RANDO Phantom Dosimetry

 A RANDO phantom and GAFCHROMIC films were used to verify dose uniformity using the STDFM TBI technique. Dose distributions were measured by placing EBT3 films between RANDO phantom sections along the AP direction in several regions of interest including head, neck, lungs, abdomen, pelvis, and the overlapped region. In addition, film strips were placed in lateral direction at the abdomen and the head to investigate lateral dose distribution. All film strips were 1.5 cm in width and cut from the same batch. Radiation was delivered to the phantom with AP/PA beams following the treatment protocol described above which adopts dynamic field edges with 3 cm leaf motion, 5.5 cm field overlap, lung shielding, and a rice bag attached to the neck. Film dosimetry procedure was performed as described above.

## 3. Results

### 3.1. Beam Intensity Compensator

**Figure 5:**
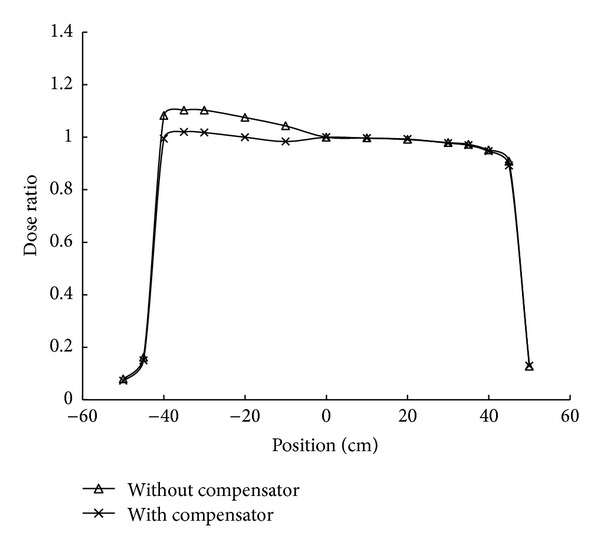
The dose profile measured at a depth of 10 cm with a gantry angle of 349° ( = 360° − 11°) and a 40 × 40-cm^2^ field. The static fields with and without a beam compensator are shown.

An oblique radiation beam results in a slanted dose distribution at depths in phantom. For a 40 × 40 cm^2^ static field, the transmission factor of a 1-mm lead sheet attached to the tray was 0.939. Dose variation of the profile at a depth of 10 cm for the 11° oblique beam without a compensator ranged from 0.96 to 1.10 between −40 and +40 cm. The dose ratio was normalized to the beam center. Dose variation with the beam compensator in place ranged from 0.95 to 1.02 as shown in Figure 5. The compensator smoothed the dose profile distribution and decreased the dose variation from 14% to 7%. 

### 3.2. Optimal Overlap Width of Dynamic Matching Fields

**Figure 6:**
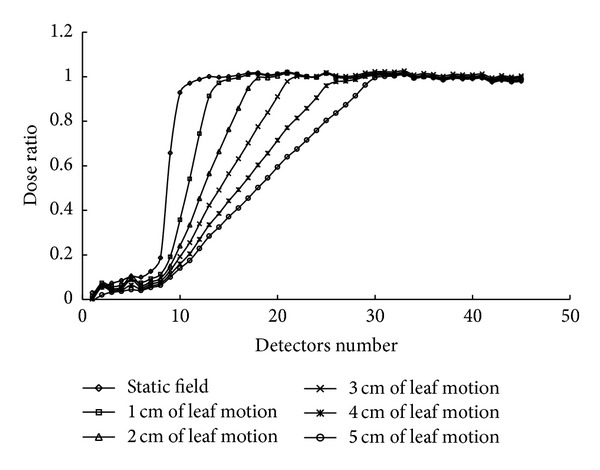
The field profiles were measured using a linear Profiler in solid water at a depth of 10 cm. A static field and dynamic field edges with various leaf motion lengths (measured at isocenter) are shown.

Figure 6 shows dose profiles near field edges of the static and the dynamic fields with leaf motion lengths of 1, 2, 3, 4, and 5 cm. These profiles were measured with a Profiler. All measurements were made at the gantry angle of 349° with the 40 × 40 cm^2^ field size at a depth of 10 cm. The horizontal axis was labeled as the number of detectors spaced 5 mm apart. The vertical axis shows the dose ratio normalized to detector No. 40, a detector far away from the field border. For dynamic field edges, the dose profiles declined gradually to the border. By comparison, the static field displayed high dose gradient at the field edge. Increases in the dynamic leaf motion length resulted in a decreased dose gradient and a broader inclined region.

**Figure 7:**
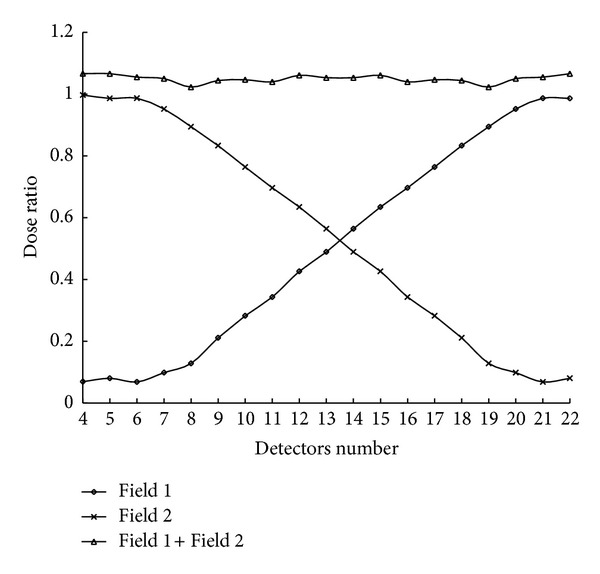
The summed dose profile at isocenter of two dynamic matching field edges with a 3 cm leaf motion and a 5.5 cm overlap at treatment distance. Field 1 was measured at depth of 10 cm and field 2 was the inverse of field 1 in position.

Figure 7 shows the combined dose profile of two matched dynamic field edges with a 5.5 cm overlap. Field 1 was measured at a depth of 10 cm with 3 cm of leaf motion, and field 2 was the reverse of the field 1 in terms of position. The dose variation in the overlapped region ranged from 102.2% to 106.1% with the dose normalized to detector No. 40.

**Figure 8:**
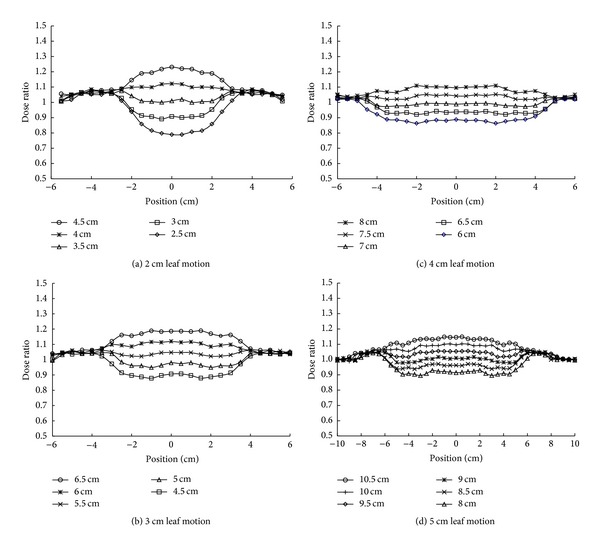
The dose distribution across the overlapped region of two dynamic matching field edges with various overlap widths was measured by a Profiler at 10 cm depth at the treatment distance. The dynamic fields involved leaf motion of (a) 2, (b) 3, (c) 4, and (d) 5 cm.

Figure 8 shows the total dose profiles of the two dynamic matching fields in the overlapped region at a depth of 10 cm for various lengths of leaf motion and different overlap widths. The horizontal axis is the distance from the center of the overlapped region. For a given leaf motion length, a broader overlap produced a higher dose distribution in the matching zone compared with a smaller overlap. To obtain a highly uniform dose distribution, longer leaf motion length was preferred.

Based on the results shown in Figure 8, optimal overlap width with the dose variation less than ±10% for different lengths of leaf motion was obtained (Table 1). The optimal overlap increased with the length of leaf motion. In addition, the range of optimal overlap was positively related to the leaf motion. For example, with 5 cm of leaf motion, the optimal width interval was 1.5 cm between 8.5 and 10.0 cm. Similarly, 3 cm of leaf motion had a 0.5 cm optimal width interval between 5 and 5.5 cm. Note that the optimal overlap changed slightly with depth (due to the change in beam divergence during leaf motion). A wider overlap has better dose uniformity, but a shorter treatment dimension. To reach a satisfied compromise between the dose uniformity and treatment dimension, 3 cm of leaf motion and 5.5 cm overlap were used in the subsequent experiments.

### 3.3. Dose Profiles in the Overlapped Region at Depths

 The dose uniformity at different depths in the overlapped region should be maintained at an acceptable level. Dose profiles were measured with strips of GAFCHROMIC films placed in the solid water at depths of 0, 5, 10, and 15 cm as shown in Table 2. The ranges of dose variation at depths of 0, 5, 10, and 15 cm were 98.4% to 103.8%, 99.3% to 105.0%, 97.9% to 102.3%, and 98.3% to 101.2%, respectively. All the doses were normalized to that of the point 5 cm away from the center of the overlapped region. This confirmed dose uniformity requirement at different depths.

### 3.4. Total Dose Profiles of Dynamic Matching Fields

**Figure 9:**
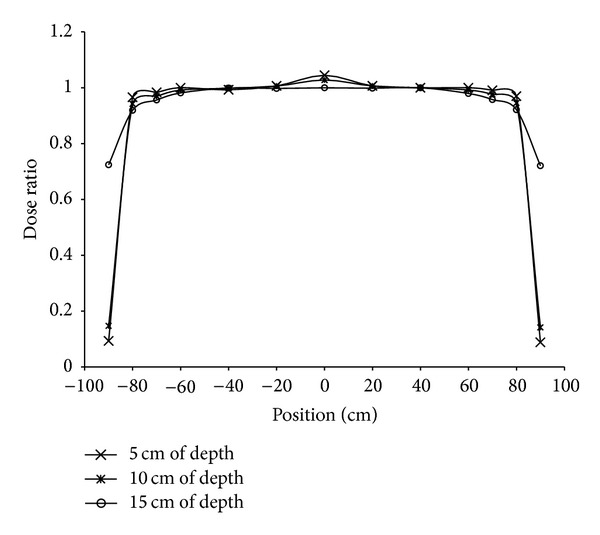
The total dose profiles of two oblique fields with matching dynamic field edges were measured along the transverse axis in a solid water phantom at depths of 5, 10, and 15 cm. The dose was normalized to that at the position 40 cm away from the center of the overlapped region.

 The dose profiles of the entire irradiated volume covered by the two oblique fields with dynamic field edges were investigated for clinical implementation.  Figure 9 shows the total dose profiles measured in the solid water phantom at depths of 5, 10, and 15 cm. The dose profiles were normalized to the position 40 cm away from the center of the overlapped region. The variations of dose profiles within ±80 cm at depths of 5, 10, and 15 cm were 96.6% to 104.4%, 94.2% to 102.8%, and 91.9% to 100.0%, respectively. The dose in the overlapped region was slightly higher due to a 5.5 cm overlap for the 3-cm leaf motion. Furthermore, the dose variation decreased with depth. This result demonstrated that the dynamic matching field edges with a beam compensator produced uniform dose distribution at various depths throughout the large treatment volume. 

### 3.5. Percentage Depth Dose in the Buildup Region

**Figure 10:**
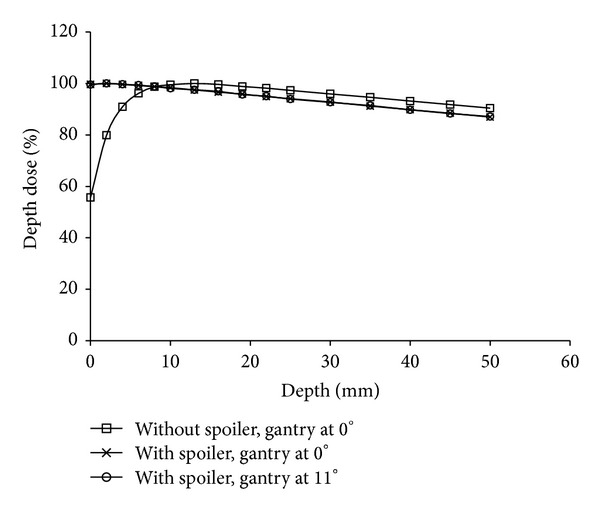
Percentage depth dose curves of a 6-MV, 40 × 40-cm^2^ photon beam measured using a Markus parallel plate ionization chamber inserted in a 20 cm thick solid water phantom on the floor. A 1 cm thick acrylic beam spoiler was placed above the floor at a distance of 30 cm. The gantry angle was set at (a) 0° without a spoiler, (b) 0° with a spoiler, and (c) 11° with a spoiler.

 A spoiler placed in front of the phantom provides extra scattered dose to the buildup region and improve the dose uniformity. The percentage depth doses of the vertical treatment field with or without the spoiler and those of the 11° oblique field with the spoiler were measured at the treatment distance, as shown in Figure 10. The use of the spoiler in the beam increased the surface dose from 57% to 99% and shifted the depth of maximum dose toward the surface from 1.3 cm to 2 mm. No significant difference was observed between the 11° oblique beam and the vertical beam with the spoiler.

### 3.6. RANDO Phantom Dosimetry

**Figure 11:**
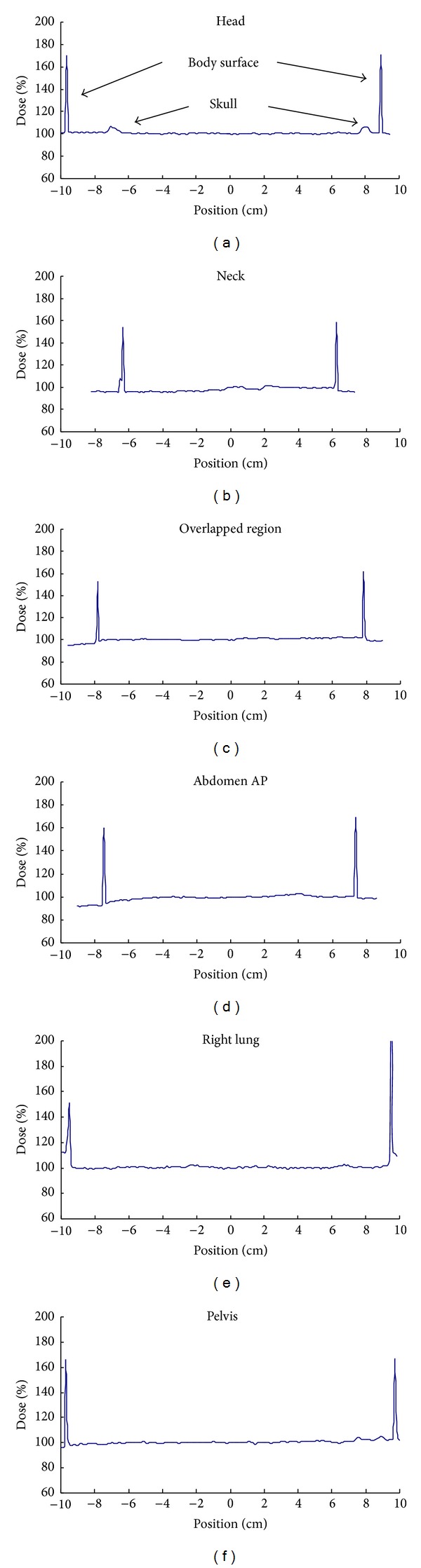
Dose distributions of step translation dynamic field-edge matching total body irradiation along the AP orientation are shown. They were measured with EBT3 film strips sandwiched between RANDO phantom sections. Measurements were made at several sites of interest, including head, neck, lungs, overlapped region, abdomen, and pelvis. The sharp peaks indicate the location of the body surface marked on the film strips. The dose was normalized to that at the midpoint of the two sharp peaks.

 The dose distribution of the STDFM TBI treatment was measured with EBT3 film strips sandwiched between the RANDO phantom sections in the AP and lateral directions.  Figure 11 shows measurements performed at several sites of interest: head, neck, lungs, overlapped region, abdomen, and pelvis. The two sharp peaks near the border of the profiles indicate the body surface marked on the film strips. Doses were normalized to the midpoint corresponding to the midline of the phantom of the two sharp peaks on the film strips. The smooth profiles between the peaks demonstrated good dose uniformity along the AP direction. No significant dose deficit was observed in the buildup region. The profile of the lung site showed a higher dose outside the phantom. This might be caused by the scattered radiation from the lung shielding above the phantom. Two obvious dose peaks corresponding to the location of the skull were also observed in the dose profile of the head. A larger numbers of electron motivated by the dense skull bone increased the dose absorption of the film by 4.6% compared to those of the surrounding soft tissues. There have been relatively few studies on the dose distribution in TBI affected by high-density bone compared with low-density lungs. The absorbed dose to bone depends on the ratio of the averaged mass energy absorption coefficient of the bone to that of the surrounding soft tissues over the photon spectrum. It is difficult to evaluate the influence of bone in TBI treatment because of difficulties involved in determining the beam spectrum, as well as the complex anatomical variations in volume, shape, and density [3].

**Figure 12:**
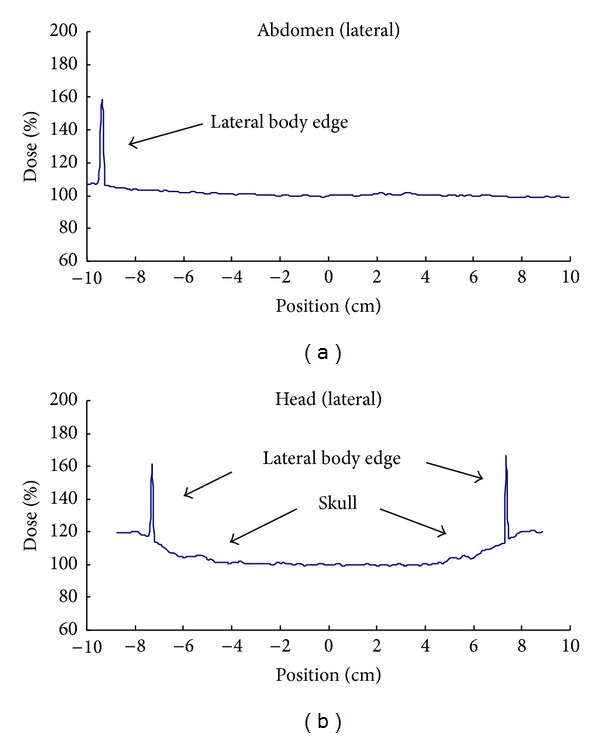
Lateral dose profiles measured with film strips positioned at abdomen and head along the lateral direction. The sharp peaks indicate location of the body surface.

To assess the complex effects of bone in TBI, we measured the attenuated dose of the head with the skull bone and that of the abdomen in the RANDO phantom with the same thickness using an ionization chamber inserted in a 6 cm thick solid water phantom at 1.5 cm of depth. The lateral separation of the head in Section 3 of the phantom was about 15 cm. The anterior-posterior separation of the abdomen in Section 25 was about 15 cm. The measurement was made using the head site in the lateral position and the abdomen site in the AP position placed above the solid water phantom with the ionization chamber inserted in it at the treatment distance. The measured dose ratio of the head site to the abdomen site was 0.93 for the same physical thickness. The results demonstrated that the head containing the skull bone attenuated radiation to a greater extent than the abdomen. Hence, bone inhomogeneity in the head should be considered when evaluating dose uniformity for body contour variation in the TBI treatment. The lateral dose profiles of the abdomen and the head show approximately 9.8% and 11.7% higher doses at the body peripherals than at the midpoint because the body thickness is reduced laterally (Figure 12).

Table 3 shows the dose ratios at the midline along the superior-inferior axis and the dose variation along the AP and lateral directions for various anatomical sites including the overlapped region. The dose ratio was normalized to the dose at the midpoint measured between the sections 27 and 28 with a separation of 17.5 cm in the AP direction. For dose variation evaluation, dose distribution was normalized to the dose at midpoint of the individual profile inside the phantom. The dose ratios at the midline of the different sites ranged from 0.945 to 1.076, except that of the lung which has a lower value of 0.561 due to the lung shielding. The dose variation along the AP direction ranged from 96.0% to 104.6% for all sites. Two higher dose peaks were observed at the skull location with a higher density. The dose variation at the overlapped region ranged from 98.5% to 102.8%, demonstrating good dose uniformity at depths within the field matching zones. 

## 4. Discussion

 This study aimed to implement a TBI treatment technique in a small treatment room with the requirement of lower radiation exposure to critical organs, especially the lungs. Due to space limitation, it is difficult to use conventional AP/PA beams to deliver a large field. Although lateral treatment using a large field with the patient sitting on a bed in a crouched position can be employed, it is difficult to keep the organ dose below the tolerance level while delivering a sufficient dose to the surrounding tissues.

This work constructed a simple beam compensator to compensate for the slanted dose profile at oblique beam incidence. It can be easily assembled using a lead sheet and no extra work is required for individual patient. Similarly, the inclined beam intensity at the abutting field edges can be created with the dynamic MLC motion and used for all patients. 

Dose uniformity in the matching zone was highly correlated with the leaf motion length of the dynamic field edges and the width of the overlapped region. A dynamic field edge with 3 cm leaf motion at the isocenter projects an approximately 6.6 cm wide region of uniform distribution at a distance of 220 cm. In theory, if a static open field has a steep square dose distribution, the linear motion of the leaves would produce a perfect inclined fluence in the field border. Consequently two complementary dynamic field edges merged with a 6.6 cm overlap should form a smooth field junction. Merging two imperfect inclined fluence distributions in the field border introduced dose inhomogeneity in the junction area. Furthermore, an additional factor affecting dose uniformity in the field junction area is the variation of the overlap widths at different depths due to the change of dynamic field-edge incident angle during beam on. We have examined the dose variation across the field junction area and determined the optimal overlap width for each dynamic field edge.

A greater leaf motion length produces a broader matching zone. Consequently, dose variation due to geometric uncertainty may be smeared and result in better dose uniformity. However, a greater field overlapped region reduces the treatment dimension. In a clinical scenario, two oblique matching fields projecting an approximately 160 cm long treatment dimension on the floor can be used to treat a 170 cm tall patient by bending the patient's legs. For taller patients whose entire body cannot be placed within the treatment field, additional conventional AP-PA fields could be given to the lower extremities which are presumably less sensitive to radiation damage.

Conventional AP/PA TBI treatment has the advantage of less body contour variation compared to the lateral beam treatment, and the midline dose variation within ±10% level is recommended. Large variation in the body contour results in excessive dose variation. Although the lower limbs do not contain sensitive organs, dose delivery should still be as accurate and as uniform as possible. Yao et al. used a simple technique to improve dose uniformity in the superior-inferior axis according to patients' contours [30]. In our RANDO phantom study of dosimetry without lower limbs, a uniform dose was achieved. A slanted beam profile due to oblique incidence provides lower radiation intensity on one side of the field. The degree of slant depends on the oblique angle. For example, a beam with an 11° oblique angle results in an approximately 14% dose difference between the two opposite sides of the profile at a depth of 10 cm in the phantom study. This suggests that an oblique incidence can serve as a virtual compensator for extremities of the body. 

The implications of our findings are limited because the dose distribution in the lateral axis cannot be modified using the current technique. From the lateral profiles measured in the RANDO phantom, the dose variations at the body periphery were 9.8% and 11.7% higher than at the midpoint for abdomen and head, respectively. Since variation in actual human body is great, it is important to recognize the possibility of dose variation along the lateral axis. 

Although dynamic TBI techniques such as dynamic beams delivered with gantry rotation and continually translating the patient during beam on can be used in a small treatment room they also introduce a broadened penumbra in the shielded region along the superior-inferior direction. If taking lung motion during breathing into account [10], the penumbra might be even larger. This might be improved by using a complicated correction method [25] or an additional process [12]. Although the lung motion during breathing makes the effect uncertain [10], the penumbra might be even larger. Therefore, fixed beam TBI techniques are preferred over the dynamic techniques for lung shielding.

Sequential field delivery TBI techniques which deliver doses with small fields sequentially might cause dose heterogeneity in the circulating blood. Molloy [31] studied this effect and concluded that the heterogeneity is acceptable in clinical practice. Our STDFM TBI technique delivers a dose with two large fields, and the effect of dose heterogeneity might be less significant than the sequential techniques. 

The over response of the dose measurement in the buildup region using a parallel plate ionization chamber could be attributed to the uncertainty of the surface dose measurement in TBI. Several authors [32, 33] corrected the effect under standard treatment condition. Yao et al. [30] measured percent depth doses using a Markus parallel plate ionization chamber and concluded that the surface dose can increase to 99% when a spoiler is used in a beam. The results (Figure 11) of our phantom study also showed that the uniform dose could be achieved in the buildup region under the TBI treatment condition. To implement TBI treatment in a small treatment room with lung shielding, several techniques have been proposed [13, 24]. These techniques have the advantage of generating a highly uniform dose distribution in three dimensions. It, however, requires additional equipment and/or complex procedures. 

## 5. Conclusions

 We examined feasibility of a simple step translation and dynamic field-edge matching TBI technique using a RANDO phantom model. The method can be used to treat patients in a small treatment room, while keeping the lung dose under the desired level without using extra equipment, complex procedures, and patient-specific compensators. 

## Figures and Tables

**Table 1:** The optimal overlap widths of the two dynamic field edges matched at a distance of 220 cm from the radiation source. When overlapped optimally, the dose variation is within ±10% at the field junction.

Leaf motion length (cm)	2	3	4	5

Optimal overlap width (cm)	3.5	5.0–5.5	6.5–7.5	8.5–10.0

**Table 2:** The dose variation in the overlapped region at various depths measured using EBT3 film strips placed in the solid water phantom slabs. Two dynamic field edges with 3 cm of leaf motion and gantry angles of 11° and 349° delivered a dose to a 20 cm-thick phantom with an overlap of 5.5 cm.

Depth (cm)	0	5	10	15

Dose variation in overlap region (%)	98.4–103.8	99.3–105	97.9–102.3	98.3–101.2

**Table 3:** The dose variation measured in a RANDO phantom using GAFCHROMIC film strips sandwiched between sections at sites of interest. The film strips were orientated along the AP direction, except for two strips along the lateral orientation at abdomen and head. The dose ratios at midpoints of profiles inside the phantom were normalized to the midpoint dose measured between sections 27 and 28. The dose variation was normalized to the midpoint of separation in body surface for individual profile.

Sites	Head	Neck	lung^1^	Overlap region	Abdomen	Abdomen	Pelvis	Head (lateral)^2^	Abdomen (lateral)^2^
Separation (cm) of body surface	18.5	13	19	15.5	14.5	17.5	19.5	18.5	14.5
Dose ratio of midpoint	0.964	1.021	0.561	1.062	1.076	1.000	0.945	0.955	1.083
Maximum dose in profile (%)	104.6	103.4	101.2	102.8	103.2	103.3	103.3	111.7	109.8
Minimum dose in profile (%)	98.9	99.1	99.3	98.5	96.0	98.4	98.0	99.7	98.4

Between phantom section	2-3	8-9	17-18	23-24	25-26	27-28	31-32	2-3	25-26

^1^With lung shielding block. ^2^Film oriented in lateral direction.
